# A Band-Aware Riemannian Network with Domain Adaptation for Motor Imagery EEG Signal Decoding

**DOI:** 10.3390/brainsci16040363

**Published:** 2026-03-27

**Authors:** Zhehan Wang, Yuliang Ma, Yicheng Du, Qingshan She

**Affiliations:** 1 School of Automation, Hangzhou Dianzi University, Hangzhou 310018, China; 23061735@hdu.edu.cn (Z.W.); 231060380@hdu.edu.cn (Y.D.); qsshe@hdu.edu.cn (Q.S.); 2 Zhejiang Provincial Key Laboratory of Brain–Computer Collaborative Intelligence Technology and Applications, Hangzhou 310018, China

**Keywords:** brain–computer interface, motor imagery, feature extraction, frequency band, Riemannian manifold, domain shift

## Abstract

Background: The decoding of motor imagery electroencephalography (MI-EEG) is constrained by core issues including low signal-to-noise ratio (SNR) and cross-session as well as cross-subject domain shift, which seriously impedes the practical deployment of brain–computer interfaces (BCIs). Methods: To address these challenges, this paper proposes a novel end-to-end MI-EEG decoding method named BARN-DA. Two innovative modules, Band-Aware Channel Attention (BACA) and Multi-Scale Kernel Perception (MSKP), are designed: one enhances discriminative channel features by modeling channel information fused with frequency band feature representation, and the other captures complex data correlations via multi-scale parallel convolutions to improve the discriminability of the network’s feature extraction. Subsequently, the features are mapped onto the Riemannian manifold. For the source and target domain features residing on this manifold, a Riemannian Maximum Mean Discrepancy (R-MMD) loss is designed based on the log-Euclidean metric. This approach enables the effective embedding of Symmetric Positive Definite (SPD) matrices into the Reproducing Kernel Hilbert Space (RKHS), thereby reducing cross-domain discrepancies. Results: Experimental results on four public datasets demonstrate that the BARN-DA method achieves average cross-session classification accuracies of 84.65% ± 8.97% (BCIC IV 2a), 89.19% ± 7.69% (BCIC IV 2b), and 61.76% ± 12.68% (SHU), as well as average cross-subject classification accuracies of 65.49% ± 11.64% (BCIC IV 2a), 78.78% ± 8.44% (BCIC IV 2b), and 78.14% ± 14.41% (BCIC III 4a). Compared with state-of-the-art methods, BARN-DA obtains higher classification accuracy and stronger cross-session and cross-subject generalization ability. Conclusions: These results confirm that BARN-DA effectively alleviates low SNR and domain shift problems in MI-EEG decoding, providing an efficient technical solution for practical BCI systems.

## 1. Introduction

Brain–computer interface (BCI) is an advanced human–computer interaction technology that translates neural activity in the brain into specific commands for controlling external electronic devices [1,2]. In the field of BCIs, electroencephalography (EEG) [3], a non-invasive method for neural signal acquisition, has been widely adopted for its portability and safety. Its applications span various fields, including driver fatigue detection [4], stroke rehabilitation [5], emotion recognition [6], and brain-controlled vehicles [7,8]. Motor imagery (MI) enables hands-free control of prostheses and wheelchairs involves users imagining the movement of specific body parts, modulating different regions of the brain’s motor cortex [9]. As an endogenous evoked EEG signal, MI is independent of specific external stimuli. Compared with SSVEP [10,11] and P300 [12], it is more suitable for controlling exoskeletons employed in patient rehabilitation [13]. However, BCI-based MI technology faces key challenges that hinder its widespread application. EEG signal has the characteristics of non-stationary and low signal-to-noise ratio (SNR) [14,15], which makes the signal easily polluted by various noises, increasing the difficulty of accurately extracting useful signals.

In recent years, various nonlinear analysis methods have emerged in brain–computer interface research, including common spatial filtering [16,17,18,19], wavelet transform [13,20,21], and detrended feature extraction [22]. Deep learning methods do not require complicated manual feature design; they can automatically and simultaneously extract spatial and temporal features from EEG signals with a simpler workflow, and have become the mainstream technique for motor imagery EEG decoding. Among them, convolutional neural networks (CNNs) such as ConvNet [23] and EEGNet [24] exhibit outstanding performance in feature extraction. Researchers have also introduced attention mechanisms to enhance feature representation, e.g., [25,26,27,28,29,30]. Derived from the fields of computer vision and natural language processing, these attention mechanisms mainly process features from the temporal domain, while the potential value of frequency domain characteristics remains to be explored.

EEG classification on high-dimensional Riemannian manifolds has recently received increasing attention to improve the performance of the EEG classification [31]. These methods [32,33,34,35] first transform features into covariance matrices and then embed the matrices into the Riemannian space for subsequent learning. They excel at preserving the intrinsic structural information of signals and enhancing the robustness of features. However, existing Riemannian geometry-based methods mainly focus on feature modeling in non-Euclidean space and fail to account for the inherent cross-session or cross-subject domain shift of EEG signals, which degrades their generalization performance in EEG decoding tasks. Domain adaptation techniques, including transfer learning [36,37], domain-adversarial training [38,39], and other methods, can alleviate the domain shift problem and effectively map the source and target domain data with different distributions. Therefore, from the perspective of integrating the advantages of both, it is necessary to develop schemes that combine Riemannian geometry methods with domain adaptation techniques.

To address the aforementioned issues, this study proposes a novel decoding method named Band-Aware Riemannian Network with Domain Adaptation (BARN-DA), which can significantly improve the decoding performance of MI-EEG signals. An innovative Band-Aware Channel Attention (BACA) module is designed in this study. This module integrates frequency band attributes while modeling channel information, possesses spectral feature representation capability, and can highlight important channels. To capture more discriminative features, this study further proposes a Multi-Scale Kernel Perception (MSKP) module, which captures fine-grained temporal dynamics at different scales through multiple parallel depthwise convolutions. In addition, this study maps the feature representations onto the Riemannian manifold, and based on the log-Euclidean metric, specially designs the Riemannian Maximum Mean Discrepancy (R-MMD) loss for aligning the feature distributions between Symmetric Positive Definite (SPD) features from the source and target domains. Comprehensive validation experiments of the BARN-DA are conducted on four public datasets. The experimental results demonstrate that the proposed method achieves excellent classification performance in the MI-EEG signal decoding task, fully verifying its technical effectiveness and practical application value. In short, the contributions of this paper can be summarized as follows:

- Propose a novel MI-EEG decoding framework, BARN-DA, which enhances the feature expression ability and thus improves the decoding performance of MI-EEG signals;
- Design an innovative BACA module that fuses frequency-domain attributes and channel information to highlight important channels;
- Develop an MSKP module, which extracts robust representations via parallel convolutions;
- The proposed R-MMD loss can directly achieve the alignment of SPD matrices across different domains, improving the model’s generalization performance.

The rest of this paper is organized as follows: Section 2 presents the related research work; Section 3 elaborates on the proposed method in detail; Section 4 describes the experimental setup; Section 5 presents the experimental results; and Section 6 summarizes the entire paper.

## 2. Related Work

Traditional MI-EEG classification methods predominantly rely on handcrafted feature extraction [40], such as power spectral density (PSD), common spatial pattern (CSP) [16], and time-domain features, which heavily depend on domain expertise and prior knowledge. The rise of deep learning has provided numerous technical solutions and new approaches for MI-EEG decoding. Schirrmeister et al. [23] proposed a ConvNet, which achieved a performance level comparable to that of the FBCSP [17] algorithm. Lawhern et al. [24] proposed EEGNet for the classification of multiple BCI paradigms and demonstrated its robust performance. Other studies have also combined band-pass filtering with neural networks. For example, FBCNet [41] creates a multi-view representation of the data through multi-band filtering uses CNN to learn spatial discriminative patterns. However, the performance gains of these strategies remain limited due to the lack of modules with strong discriminative power.

The attention mechanism enables the model to focus on the most relevant time segments or feature dimensions for the task. Miao et al. [42] proposed LMDA-Net, which integrates channel attention modules and depth attention modules specifically designed for EEG signals. Wimpff et al. [29] systematically compared the impact of different channel attention mechanisms on motor imagery decoding. Qin et al. [28] proposed ETCNet, introducing an Efficient Channel Attention module before feature extraction to enhance the extraction capability of channel-specific features. Han et al. [43] proposed SST-DPN and designed a lightweight spatial–spectral attention mechanism to capture powerful spatial–spectral features, while these mechanisms have achieved certain results in improving model performance, few studies have considered the frequency characteristics of EEG features and incorporated them into the module structure.

Riemannian geometry methods, which can effectively handle complex geometric data structures, are widely applied in the field of BCIs. Based on machine learning methods, Fang et al. [44] proposed using filter banks and the Riemannian tangent space, combined with SVM for classification. Jin et al. [45] proposed combining Riemannian geometry with sparse optimization and introducing the Dempster–Shafer theory for multi-time window feature fusion. In addition, Riemannian geometry has also been combined with deep learning methods. Tensor-CSPNet [32] converts EEG signals into SPD matrices, captures spatial patterns through BiMap and Riemannian batch normalization layers, and extracts temporal features using CNNs. Graph-CSPNet [33] extends graph CNNs to the SPD manifold. Liang et al. [34] adopted the second-order pooling method to aggregate the covariance features of EEG signals and then leveraged Riemannian geometry learning to map these features. Shi et al. [35] combined Riemannian geometry with neural networks, adopting a multi-branch structure, which improved decoding performance.

Domain adaptation reduces the distribution discrepancy between the source domain and the target domain by leveraging source domain information and learns domain-invariant features to improve the MI-EEG classification performance of the target domain. Many methods based on the idea of domain adaptation have been proposed. Hong et al. presented DJDAN [46], which adopts adversarial learning, aligns inter-domain distributions via global and local discriminators, and introduces dynamic adversarial factors to adjust alignment weights. She et al. developed DAWD [38], which measures inter-domain discrepancies based on the Wasserstein distance and achieves distribution alignment by combining gradient penalty with adversarial learning. Zhao et al. introduced DRDA [47], which constructs a deep representation space through the joint optimization of three modules and introduces center loss to reduce intra-domain non-stationarity. GAT [48], proposed by Song et al., adopts spatio-temporal convolution and attention adapters and guides feature transfer with a dual-alignment mechanism. For better cross-domain generalization, Zhong et al. established EEG-DG [49], which constructs a generalization model using multiple source domains and obtains invariant features by jointly optimizing distributions. In general, traditional domain adaptation methods are only applicable to the Euclidean space, while this study introduces the log-Euclidean metric to define the manifold distance, thus enhancing the alignment of feature distributions on the manifold.

To synthesize the differences among existing methods and explicitly demonstrate the innovations of BARN-DA, we conduct a comprehensive comparison across the core dimensions discussed earlier. The results are summarized in Table 1.

## 3. Method

**Figure 1:**
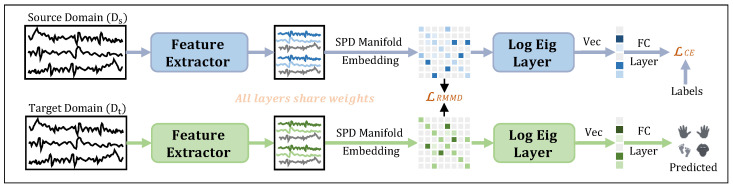
Overall framework structure of BARN-DA.

Figure 1 presents the system architecture of the proposed BARN-DA. The EEG data from the source and target domains are fed into the network separately, where they successively undergo feature extraction, SPD manifold embedding, and LogEig layer processing. Then, the data are vectorized and combined with the fully connected layer to yield the final output. Details of each module will be elaborated one by one in the following sections.

### 3.1. Feature Extractor

**Figure 2:**
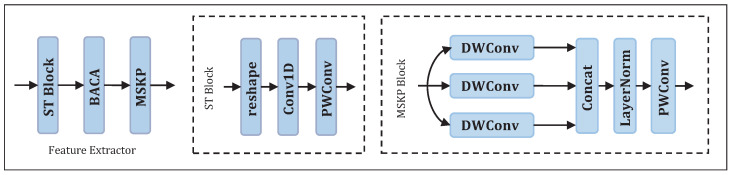
Structure of the feature extractor.

The feature extractor is designed to extract discriminative features from the input EEG signals. As shown in Figure 2, the feature extraction method proposed in this paper is specifically divided into three modules, namely the spatial-temporal block, the custom-designed BACA module, and the MSKP module.

#### 3.1.1. Spatial-Temporal Block

EEG signals are characterized by high temporal resolution. Therefore, capturing their fine-grained local temporal features is the key to improving the performance of motor imagery recognition. Inspired by the research of Han et al. [43], this paper adopts lightweight convolution to extract the temporal features of EEG signals and combines it with pointwise convolution to obtain spatial representations. For the input EEG signal $X\in R^{C\times T}$ (where *C* denotes the number of EEG channels and *T* represents the length of the time series), dimension reshaping is first performed to convert it into the format of $R^{C\times 1\times T}$. Subsequently, one-dimensional convolution is executed using a shared convolution kernel $W\in R^{D\times 1\times K}$ (where *D* is the number of convolution kernel groups and *K* is the size of the convolution kernel in the time domain). Through the parameter sharing mechanism, this process efficiently captures the universal temporal rhythmic features of MI-EEG signals while reducing the number of model parameters. The dimension of the output features is expanded to CD, and channel feature fusion is implemented by combining with pointwise convolution. The overall process can be described by the following formula:

(1) $$\begin{array}{cc} \; & X_{reshaped}=Reshape\left(X\right)\in R^{C\times 1\times T},\\ \; & X_{temporal}=Conv1D\left(X_{reshaped},W\right)\in R^{CD\times T},\\ \; & X_{out}=PWConv1D\left(X_{temporal}\right)\in R^{F\times T}, \end{array}$$

where *F* represents the final output feature dimension.

#### 3.1.2. BACA Module

**Figure 3:**
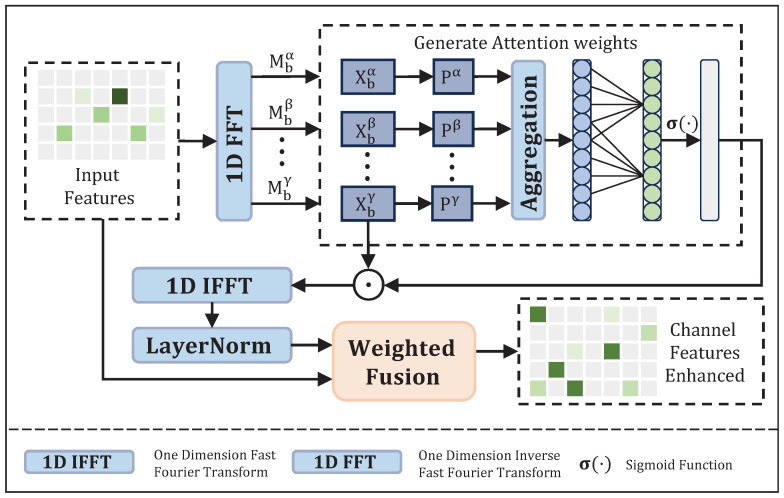
The architecture of BACA module.

EEG signals possess distinct physiological functions and unique cognitive associations across different frequency bands. Inspired by the research of [50], we propose a novel BACA module, whose structure is illustrated in Figure 3. This module innovatively incorporates frequency-band prior knowledge into the design of the channel attention mechanism, achieving targeted channel-wise feature enhancement.

Assume the input signal is $x\in R^{F\times T}$, where the frequency band set is denoted as B, covering the δ, θ, α, β, and γ bands of EEG signals, and the total number of frequency bands is recorded as |B|. First, the time-domain signal is mapped to the frequency domain using the Fast Fourier Transform (FFT) to separate frequency components, which is described as:

(2) X=F(x).

Subsequently, frequency-band filtering is performed on the frequency-domain features to extract specific physiological sub-features:

(3) $$X_{b}=X⊙M_{b},$$

where b∈B denotes a specific frequency band; $M_{b}$ is a binary indicator mask for frequency band *b*, which takes a value of 1 only within the corresponding frequency interval.

Prior to the calculation of attention weights, we first compute the energy intensity of each frequency band and perform mean aggregation on the power of each frequency band to obtain channel-level global energy features. Subsequently, 1D convolution is applied to capture the local dependencies between channels, and channel attention weights are finally generated. The mathematical expression is as follows:

(4) $$W_{c}=Sigmoid\left(Conv1D\left(\frac{1}{\left|B\right|}\sum_{b\in B}\left(\sum_{n=1}^{N}{\left|X_{b}\left(n\right)\right|}^{2}\right)\right)\right),$$

where *N* represents the dimension of the frequency domain. The calculation method for the size of the convolution kernel adopts the idea of ECANet [51], with the expression as follows:

(5) $$k={\left\lfloor \frac{\log_{2}\left(F\right)+b}{\gamma }\right\rfloor }_{\mathrm{odd}},$$

where *F* denotes the number of channels, and γ and *b* are set to 2 and 1, respectively.

The frequency-domain features of each frequency band are weighted and summed according to the channel attention weights to achieve channel feature enhancement, with the mathematical expression:

(6) $$X_{fused}=\sum_{b\in B}X_{b}⊙W_{c}.$$

The fused frequency-domain features are mapped back to the time domain via the Inverse Fast Fourier Transform (IFFT), and then layer normalization is applied to eliminate amplitude differences, thereby stabilizing the model training process. To avoid feature distortion, we introduce a learnable parameter α with a value range of (0, 1) for adaptive fusion of the enhanced features obtained through the aforementioned processing and the original features. This parameter automatically adjusts during training to flexibly regulate the fusion ratio between the enhanced features and the original features.

#### 3.1.3. MSKP Block

To further improve the robustness and multi-scale representation capability of temporal feature extraction, an MSKP block is proposed in this paper. By deploying parallel depthwise convolution branches with different kernel sizes, this block enables sufficient capture and fusion of features across different time windows. The input EEG features are simultaneously fed into three parallel depthwise convolution branches with kernel sizes of k=3, k=5 and k=7, respectively. The resulting features are concatenated along the channel dimension to form multi-scale aggregated features. Layer normalization is adopted to regularize the feature distribution, and combined with pointwise convolution, it compresses the channel dimension and conducts feature mapping to restore the channel number to the original dimension *F*, thus extracting discriminative features. The calculation process of this block is formally expressed as:

(7) $$\begin{array}{cc} \; & X_{i}=DWConv1d\left(X,k_{i}\right),\;k_{i}\in \left\{3,5,7\right\},\;i=1,2,3,\\ \; & X_{cat}=Concat\left(X_{i}\right)\in R^{3F\times T},\\ \; & X_{out}=PWConv1d\left(LayerNorm(X_{cat})\right)\in R^{F\times T}. \end{array}$$

### 3.2. Riemannian Geometry

Recent approaches to BCI classification have notably shifted from traditional Euclidean metrics to employing Riemannian geometry, better reflecting the complex data structures [52]. This paper proposes embedding features into the Riemannian manifold and utilizing the Log-Euclidean transform to project the SPD matrices on the manifold onto the Riemannian tangent space, thereby achieving the transformation from the nonlinear manifold space to the linear Euclidean space.

#### 3.2.1. SPD Manifold Embedding

First, we project the extracted features onto the SPD manifold by constructing a covariance-like matrix with centering and regularization. The SPD matrix *P* can be computed by:

(8) $$P=XC_{m}X^{T}+\epsilon I_{c},$$

where $C_{m}$ is the centering matrix, $I_{c}$ is the identity matrix, and ϵ is a regularization term. The SPD manifold is a typical Riemannian manifold, and the geodesic distance $\delta_{R}\left(P,P^{\prime }\right)$ represents the length of the shortest path between points *P* and $P^{\prime }$ on the manifold. The logarithmic Euclidean metric is used to capture the nonlinear distance between two SPD matrices along the geodesic, avoiding the high computational cost of other Riemannian metrics. Its geodesic distance is defined as:

(9) $$\delta_{R}\left(P,P^{\prime }\right)={∥\log\left(P\right)-\log\left(P^{\prime }\right)∥}_{F}$$

where ${∥\cdot ∥}_{F}$ is the Frobenius norm of the matrix.

#### 3.2.2. LogEig Layer

If the SPD matrices on the non-Euclidean Riemannian manifold are directly treated as ordinary Euclidean matrices, the inherent geometric structure of the data will be damaged, resulting in the loss of feature information. Therefore, it is necessary to map the SPD matrices to their corresponding tangent spaces while ensuring that this mapping process is differentiable to meet the gradient propagation requirements for end-to-end training of neural networks. Thus, this paper adopts the matrix logarithm mapping:

(10) $$X=U\log\left(\Sigma \right)U^{T},$$

where *P* is an SPD matrix, *U* is the eigenspace of *P*, and Σ is the diagonal matrix of eigenvalues of *P*.

The aforementioned differentiable mapping process provides a prerequisite for backpropagation and, thus, can be integrated into the backpropagation pipeline of the neural network. The specific formula is expressed as follows:

(11) $$\frac{\partial L^{\left(l\right)}}{\partial P}=U\left(L⊙\left(U^{T}\frac{\partial L^{\left(l+1\right)}}{\partial X}U\right)\right)U^{T},$$

where $L^{\left(l\right)}$ is the loss at layer *l*, *L* is the Loewner matrix, which is constructed from the eigenvalues of the SPD matrix and serves to quantify the gradient transfer relationship of matrix logarithmic mapping. It is specifically described as:

(12) $$L_{ij}=\left\{\begin{array}{cc} \frac{\log\lambda_{i}-\log\lambda_{j}}{\lambda_{i}-\lambda_{j}}, & \lambda_{i}\neq \lambda_{j}\\ \frac{1}{\lambda_{i}}, & \lambda_{i}=\lambda_{j} \end{array}\right.,$$

where $\lambda_{i}$ and $\lambda_{j}$ denote the *i*-th and *j*-th eigenvalues of the SPD matrix *P*, respectively.

#### 3.2.3. Vectorization

The vectorization module is designed to convert the symmetric matrix output by the LogEig Layer into a one-dimensional feature vector. Based on the symmetry of the output matrix, this module only extracts the elements in the upper triangular region, including the diagonal, to achieve vectorization. For the input symmetric matrix $X\in R^{F\times F}$, its elements are denoted as $X_{ij}$ (i,j=1,2,…,F), which satisfy the symmetry property $X_{ij}=X_{ji}$. The vectorization function is defined as:

(13) $$v=vec\left(X\right)\in R^{\frac{F(F+1)}{2}}.$$

The output feature vector is further fed into the classification layer to realize the final classification prediction and result output:

(14) $$\hat{y}=Softmax\left(W\cdot v+b\right),$$

where *W* and *b* represent the weight matrix and bias term of the fully connected layer, respectively.

### 3.3. Riemannian Geometry-Based Domain Adaptation

Domain adaptation serves as an effective approach to mitigate the distribution divergence between EEG features derived from the source domain and those from the target domain. However, features on the Riemannian manifold fail to satisfy the linearity assumption of Euclidean space, making it difficult to directly apply traditional alignment methods. To address this issue, this paper proposes a domain alignment framework leveraging Riemannian Maximum Mean Discrepancy (R-MMD). The Log-Euclidean metric is introduced to simplify geometric operations on the manifold, transforming the non-Euclidean distribution discrepancy on the manifold into a Euclidean optimization problem in the tangent space.

**Figure 4:**
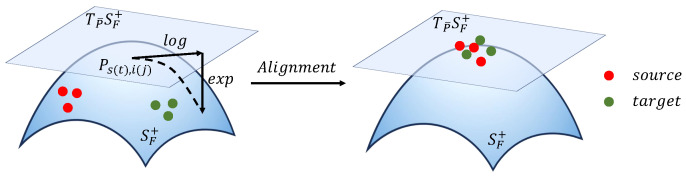
Domain alignment based on Riemannian geometry.

Let the source domain sample set be $D_{s}={\left\{P_{s,i}\right\}}_{i=1}^{m_{s}}$ and the target domain sample set be $D_{t}={\left\{P_{t,j}\right\}}_{j=1}^{m_{t}}$, where $P_{s,i},P_{t,j}\in S_{F}^{+}$ are *F*-order SPD matrices. Under the Log-Euclidean metric framework, the tangent space at any point on the Riemannian manifold $S_{F}^{+}$ is linearly isomorphic to the symmetric matrix space. As shown in Figure 4, all SPD matrices from the source and target domains are projected onto this tangent space $T_{\bar{P}}S_{F}^{+}$ (where $\bar{P}$ denotes a fixed reference point on $S_{F}^{+}$) via logarithmic mapping.

To quantify and reduce the domain shift, the R-MMD loss is introduced as the alignment constraint. Different from Euclidean MMD, the kernel function of R-MMD based on the Log-Euclidean metric is directly defined by the manifold distance, which can better preserve the geometric structural information of SPD matrices. The Gaussian kernel function based on the Log-Euclidean distance is defined as:

(15) $$k\left(P,P^{\prime }\right)=\exp\left(-\frac{\alpha }{2}{∥\log\left(P\right)-\log\left(P^{\prime }\right){∥}_{F}}^{2}\right),$$

where α>0 is a kernel parameter related to the kernel width, which balances the smoothness and discriminability of the kernel function. Leveraging the properties of the Reproducing Kernel Hilbert Space (RKHS), the R-MMD loss can be expressed as:

(16) $$L_{R-MMD}={∥\frac{1}{m_{s}}\sum_{i=1}^{m_{s}}\phi \left(P_{s,i}\right)-\frac{1}{m_{t}}\sum_{j=1}^{m_{t}}\phi \left(P_{t,j}\right)∥}_{H}^{2},$$

where ϕ(·) is the feature mapping corresponding to the kernel function, satisfying $k\left(P,P^{\prime }\right)=\langle \phi \left(P\right),\phi \left(P^{\prime }\right)\rangle$. Finally, the domain alignment loss is combined with the classification loss to achieve the unification of domain invariance and discriminability of features:

(17) $$L=L_{CE}+\lambda L_{R-MMD},$$

where $L_{CE}$ is the cross-entropy loss, and λ>0 is the balancing parameter that coordinates the optimization weights between domain alignment and classification task performance.

### 3.4. Performance Metric

To quantitatively evaluate the effectiveness of the proposed method, classification accuracy is adopted as the primary performance metric, which is defined as follows:

(18) $$Accuracy=\frac{TP+TN}{TP+TN+FP+FN}$$

where TP, TN, FP, and FN denote the numbers of true positives, true negatives, false positives, and false negatives, respectively.

To further validate the statistical significance of the experimental results and ensure that the performance improvements of the proposed method are not due to chance, the Wilcoxon signed-rank test is conducted to compare the classification accuracy scores between the proposed method and each baseline model. The Wilcoxon test is a non-parametric statistical hypothesis test, which is robust and suitable for comparing paired samples without assuming the normality of the data. The test statistic *W* is calculated based on the positive and negative ranks of the absolute differences between pairs of observations $\left(d_{i}=x_{i,proposed}-x_{i,baseline}\right)$:

(19) $$W=\min\left(\sum_{i=1}^{n}R_{i}^{+},\sum_{i=1}^{n}R_{i}^{-}\right)$$

where $R_{i}^{+}$ and $R_{i}^{-}$ represent the ranks of the positive and negative differences $d_{i}$, respectively, and *n* is the number of paired observations. A *p*-value less than a predefined significance level (p<0.05) is used to determine whether the difference in performance is statistically significant.

## 4. Experiments

This section introduces the datasets used to validate the model and elaborates on the relevant details of the experiments and parameter settings.

### 4.1. Datasets

Four MI-EEG public datasets were utilized in this study: BCIC IV 2a [53], BCIC IV 2b [54], BCIC III 4a [55], and SHU [56]. The basic information of these datasets is shown in Table 2. And the datasets are described in detail as follows:

- BCIC IV 2a: These datasets contain EEG recordings of nine participants performing four motor imagery tasks, with the task types being left hand, right hand, both feet, and tongue movements. Each participant completed two experimental sessions, and the data were sampled at 250 Hz using 22 EEG electrodes and 3 EOG electrodes. In this study, a 4 s time window was adopted, with a time range of 2–6 s. For cross-session experiments, the first session was used as the training set, and the second as the test set. For cross-subject experiments, leave-one-subject-out (LOSO) strategy is used for evaluation.
- BCIC IV 2b: This is a visually evoked potential EEG dataset for left-hand and right-hand motor imagery, collecting EEG and EOG signals from nine subjects. The data are divided into two categories: no visual feedback and visual feedback, corresponding to the first two sessions and the last three sessions, respectively, with a sampling frequency of 250 Hz. In this study, a time window of 3–7 s was selected. For cross-session experiments, the first three sessions were used as the training set, and the last two as the test set. For cross-subject experiments, the LOSO strategy is used for evaluation.
- BCIC III 4a: The dataset contains EEG recordings from five healthy subjects performing motor imagery of the right hand and right foot. Each subject completed four initial feedback-free sessions. EEG data were acquired using 118 electrodes based on the extended international 10–20 system, with a raw sampling rate of 1000 Hz; in this study, data were downsampled to 100 Hz. Visual cues for motor imagery lasted 3.5 s, with a random relaxation interval of 1.75–2.25 s between cues. Each subject had 280 trials. These dataset was used for cross-subject experiments in our study, with a LOSO validation scheme.
- SHU: These datasets contain EEG recordings from 25 healthy subjects for the motor imagery task of left and right hand grasping. Each subject completed five independent feedback-free sessions at an interval of 2–3 days. EEG data were acquired with 32 electrodes based on the international 10–10 system at a raw sampling rate of 250 Hz, and band-pass filtered at 0.5–40 Hz after acquisition. Visual and auditory cues for motor imagery lasted 4 s per trial, with 90–100 trials per session. In this study, the dataset was used for cross-session experiments, with the first three sessions as the training set and the last three as the test set.

### 4.2. Experiment Setup

All experiments were implemented using Python 3.10, the PyTorch 2.6.0 deep learning framework, and CUDA 12.6 for acceleration. All model training and inference were performed on a computer equipped with a single NVIDIA RTX 2060 GPU. Training hyperparameters are summarized in Table 3, and the model structure hyperparameters for the four datasets are detailed in Table 4.

To verify the superiority and generalization capability of the proposed method in this study, a total of 10 existing models were selected for systematic comparison. These models cover different types of network architectures and have been widely applied and validated in related tasks, namely ConvNet [23], EEGNet [24], DRDA [47], DJDAN [46], ATCNet [27], DAWD [38], GAT [48], CTNet [30], EEG-DG [49], and SST-DPN [43]. For comparative models with accessible open-source code, the experiments were conducted in the identical training environment as specified in this paper to carry out performance evaluation.

## 5. Results

This section presents the performance of the proposed method on four datasets. Meanwhile, the results of the ablation study verify the effectiveness of the designed modules. In addition, the impact of variations in several key parameters on the model performance is analyzed in detail.

### 5.1. Cross-Session Results

The cross-session classification accuracy results of the proposed method and various existing methods on all subjects of three datasets are presented in Table 5, Table 6, and Table 7, respectively. In these tables, bold font marks the optimal results among all comparative methods, while underlined font indicates the suboptimal results.

In terms of overall average accuracy, BARN-DA consistently outperforms all comparative methods across the three datasets. On the BCIC IV 2a dataset, BARN-DA achieves an average accuracy of 84.65%, representing a 2.86% improvement over the second-best method, EEG-DG, and a substantial 23.15% increase over ConvNet. On the BCIC IV 2b dataset, BARN-DA attains an average accuracy of 89.19%, surpassing the second-best method, EEG-DG, by 2.07% and the conventional method, EEGNet, by 5.51%. On the SHU dataset, which involves 25 subjects, a larger sample size, and more complex individual differences, BARN-DA still achieves an average accuracy of 61.76%, outperforming the second-best method, CTNet, by 3.35% and ConvNet by 3.95%. These results confirm that the proposed model can effectively adapt to diverse data distribution characteristics and exhibits strong generalization ability.

From the perspective of statistical significance, the *p*-values in the table further validate the reliability of the performance improvements achieved by BARN-DA. On the BCIC IV 2a dataset, BARN-DA shows significant differences (p<0.05) from 9 out of 10 comparative methods, indicating that the performance gain is not attributable to random factors. On the BCIC IV 2b dataset, BARN-DA exhibits significant differences (p<0.05) from 7 out of 10 methods, further demonstrating the statistical reliability of its advantages. On the largest SHU dataset, BARN-DA presents significant differences (p<0.05) from all 5 comparative methods, suggesting that the performance improvement of BARN-DA remains statistically robust even in scenarios with a large number of subjects and pronounced individual differences.

Regarding the single-subject performance distribution, BARN-DA demonstrates excellent inter-subject robustness. Among the 9 subjects in BCIC IV 2a, BARN-DA achieves the best accuracy for 4 subjects and the second-best accuracy for 2 subjects. Notably, on subject S02, where most methods perform poorly, BARN-DA still reaches 69.10%, significantly outperforming the baseline method. Among the 9 subjects in BCIC IV 2b, BARN-DA achieves the best results for 3 subjects and the second-best for 2 subjects, with a peak accuracy of 98.12% on subject S05. On the SHU dataset with 25 subjects, BARN-DA attains the best results for 13 subjects and the second-best for 2 subjects. Even on subjects with relatively low overall accuracy, BARN-DA maintains competitive performance.

**Figure 5:**
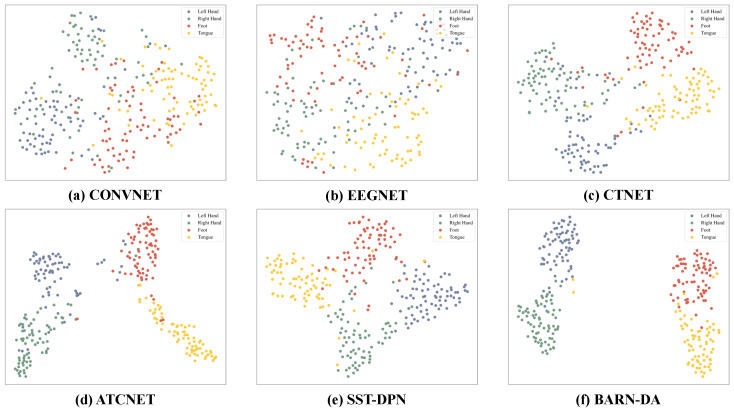
Distribution of features of different models under four types of motor imagery tasks for Subject 7 in dataset 2a.

We visualized the feature distribution extracted by the model by reducing its dimensionality to a 2D plane using the t-SNE algorithm [57], and the results are shown in Figure 5. Compared with other models, the feature distribution of the proposed BARN-DA is the clearest: the feature points of the four categories form independent clusters, respectively, with distinct inter-category boundaries, demonstrating stronger feature discriminability, which indicates that the model can learn more discriminative feature representations.

### 5.2. Cross-Subject Results

The cross-subject generalization capability of the model is a core indicator for practical BCI applications. In this study, the cross-subject performance of the proposed BARN-DA model is comprehensively validated on three public datasets, namely BCIC IV 2a, BCIC IV 2b, and BCIC III 4a, with the experimental results presented in Table 8, Table 9, and Table 10, respectively.

On the BCIC IV 2a dataset, BARN-DA achieves an average cross-subject accuracy of 65.49%, which is 3.49% higher than that of CTNet. From the perspective of single-subject results, BARN-DA attains the highest accuracy on six subjects. Notably, the accuracy for subject S03 is particularly prominent, reaching 82.29%, which far exceeds that of other comparative models. Statistical significance test results indicate that the performance differences between BARN-DA and most of the other comparative models are statistically significant (p<0.05), which statistically confirms the superiority of BARN-DA in complex four-class cross-subject tasks.

On the BCIC IV 2b dataset, the cross-subject advantage of BARN-DA is consistently maintained, with an average accuracy of 78.78%, exceeding the suboptimal model CTNet by 2.28%. In terms of single-subject performance, BARN-DA achieves the optimal results on six subjects. Compared with other comparative models, BARN-DA not only delivers a higher average accuracy but also exhibits a more balanced performance distribution across different subjects, verifying its adaptive learning capability for EEG features of diverse individuals.

To further validate the model’s generalization ability, cross-subject experiments are also conducted on the BCIC III 4a dataset. The results show that BARN-DA achieves an average accuracy of 78.14% on these datasets, substantially outperforming the suboptimal model ConvNet with an improvement margin of 8.43%. For single-subject results, BARN-DA attains the highest accuracy on all five subjects (aa, al, av, aw, ay), among which the accuracy for subject al reaches 95.71%, demonstrating strong cross-subject adaptability. Statistical test results also confirm that the performance differences between BARN-DA and the comparative models are statistically significant (p<0.05), fully proving that the model is not limited to specific datasets but possesses universal advantages in BCI cross-subject tasks of varying types and difficulty levels.

### 5.3. Ablation Results

To verify the independent contributions and synergistic effects of each core component of the BARN-DA model, this study designed a series of systematic ablation experiments. As shown in Table 11, all ablation variants with single or multiple components removed exhibited a decrease in model accuracy, which demonstrates the necessity of the design of each module.

Specifically, after removing the BACA module, the cross-session accuracy of the model decreases by 2.28% on the BCIC IV 2a dataset and 1.04% on the BCIC IV 2b dataset. In cross-subject scenarios, the influence of this module is more significant on BCIC IV 2a, with a drop of 2.18% (p=0.0020), while its contribution is relatively limited on BCIC IV 2b and BCIC III 4a. This indicates that the BACA module effectively improves the utilization efficiency of frequency-domain information and enhances the class discriminability of features by integrating frequency band prior knowledge and channel importance weights.

The removal of the MSKP module results in the most significant performance degradation. The accuracy decreases by 5.91%, 1.53%, 7.25%, and 3.69% on the BCIC IV 2a cross-session, BCIC IV 2b cross-session, BCIC IV 2a cross-subject, and BCIC IV 2b cross-subject datasets, respectively, with all *p*-values less than 0.01, indicating high statistical significance. Even on the SHU cross-session dataset, the accuracy drops by 2.02%, approaching the significance level (p=0.0564). This verifies that the module can enhance the robustness of the model through parallel multi-scale feature extraction.

Notably, the simultaneous removal of the BACA and MSKP modules leads to the largest performance decline, with accuracy reductions of 8.03%, 5.78%, 12.98%, and 5.34% in the BCIC IV 2a cross-session, BCIC IV 2b cross-session, BCIC IV 2a cross-subject, and BCIC IV 2b cross-subject experiments, respectively, and all *p*-values are less than 0.01. This highlights the synergistic effect of the frequency-domain attention mechanism and the multi-branch convolution structure, which jointly enhance the representation capability of the model.

In addition, the removal of the $L_{R-MMD}$ loss also leads to consistent performance degradation of the model on all datasets, with a reduction range from 1.09% to 8.07%, and the *p*-values of all datasets are less than 0.05, indicating statistical significance. This result confirms that the $L_{R-MMD}$ can achieve distribution alignment between the source domain and the target domain on the Riemannian manifold, effectively alleviate the performance loss caused by inter-domain differences, and further improve the recognition performance of cross-domain EEG signals.

### 5.4. Complexity Analysis

**Figure 6:**
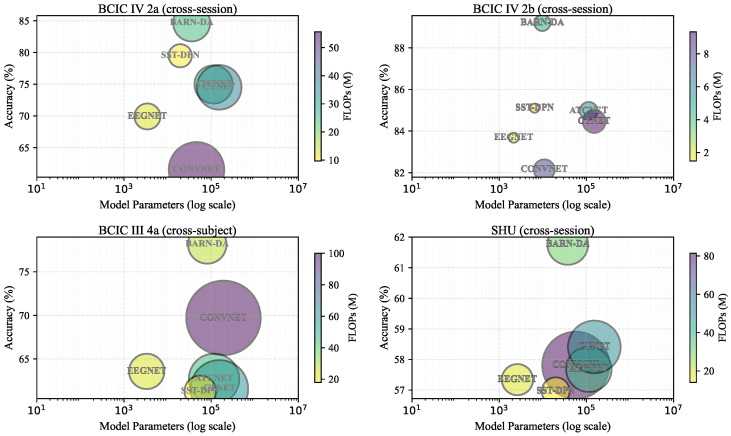
Bubble chart comparison of model performance, parameter count, and computational overhead across different datasets.

The analysis of model parametric complexity can well reflect the theoretical computational load of different models, and the relevant results are shown in Table 12. In this paper, two key model efficiency metrics are analyzed theoretically: the number of floating-point operations (FLOPs) is adopted to quantify the computational cost, and the number of parameters (Params) is used to measure the scale of trainable parameters of the model. It can be seen from the theoretical calculation results that the proposed model maintains moderate computational overhead on all datasets. On the BCIC IV 2a dataset, BARN-DA has 35,622 parameters and 24.55M FLOPs, which are much lower than those of ATCNet (113,732 parameters and 26.36M FLOPs) while slightly higher than those of EEGNet (3444 parameters and 12.20M FLOPs). As shown in Figure 6, a bubble chart is adopted to intuitively visualize the relationship among model performance, parameter count, and computational overhead across different datasets. It can be seen that the proposed method achieves a favorable balance between model performance and computational efficiency.

In addition, to more intuitively estimate the actual time consumption of the proposed method, we further tested and counted the training time and test time of the model. All-time metrics are the average values of multiple measurements for a single subject completing the full training and testing process in each dataset, which can more realistically reflect the actual runtime performance of different models on specific hardware platforms. The comparison results show that BARN-DA has more efficient training speed and faster inference speed than complex models such as ATCNet with a larger number of parameters. On the BCIC IV 2b dataset, the training time and test time of ATCNet are 343.12 s and 0.675 s, respectively, while those of BARN-DA are only 132.31 s and 0.068 s, representing reductions of approximately 61.4% and 89.9%, respectively. On the SHU dataset, the training time of ATCNet is 307.85 s, while that of BARN-DA is reduced to 193.16 s, showing a significant improvement in efficiency. Even compared with lightweight models such as EEGNet and SST-DPN, BARN-DA still maintains certain competitiveness in runtime efficiency. Taking the BCIC III 4a dataset as an example, the test times of EEGNet and SST-DPN are 0.024 s and 0.056 s, respectively, and the test time of BARN-DA is 0.070 s. Although slightly increased, BARN-DA significantly outperforms these methods in classification accuracy. The above comprehensive advantages demonstrate that BARN-DA not only exhibits excellent theoretical efficiency but also has important application value in practical deployment scenarios with high real-time requirements or limited resources.

### 5.5. Parameter Experiments

**Figure 7:**
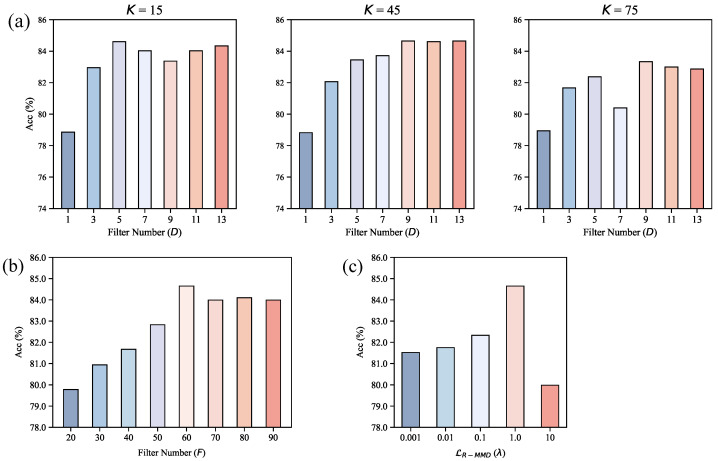
Parameter sensitivity analysis results on the BCIC IV 2a dataset (cross session). (**a**) Effect of *K* and *D* on accuracy. (**b**) Effect of *F* on accuracy. (**c**) Effect of $L_{R-MMD}$ weight λ on accuracy.

**Figure 8:**
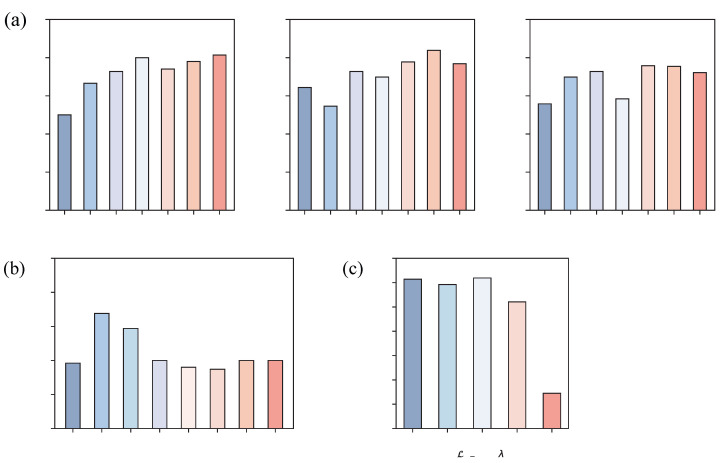
Parameter sensitivity analysis results on BCIC IV 2b dataset (cross session). (**a**) Effect of *K* and *D* on accuracy. (**b**) Effect of *F* on accuracy. (**c**) Effect of $L_{R-MMD}$ weight λ on accuracy.

In this section, we carefully evaluate the influence of several key hyperparameters on the classification performance of the model. Experiments are conducted on two representative datasets, namely BCIC IV 2a and BCIC IV 2b, with the results shown in Figure 7 and Figure 8, respectively.

The coupling effect of the kernel size *K* and the number of filters *D* of the temporal convolution in the spatial-temporal block on model performance is systematically analyzed. On both datasets, the model accuracy exhibits an overall trend of rising significantly first and then stabilizing as *D* increases, indicating that *D* plays a crucial role in determining the temporal feature extraction capability of EEG signals. Specifically, when *D* is small, the limited feature channels cannot cover the fine-grained temporal dynamics of EEG signals, resulting in low classification accuracy. As *D* increases, the feature space is expanded, enabling effective modeling of the complex temporal variations underlying electroencephalogram responses, and thus the accuracy is rapidly improved. When *D* exceeds a certain range, the feature space has already covered the main temporal patterns, and further increasing *D* only introduces redundant dimensions without bringing additional discriminative gains. In terms of the selection of *K*, the two datasets show a consistent trend that K=45 is significantly superior to K=15 and K=75. Under the condition of D=9 on the BCIC IV 2a dataset, the accuracy of K=45 reaches 84.65%, which is higher than that of K=15 and K=75. On the BCIC IV 2b dataset, K=45 also maintains the best performance under most values of *D*. This indicates that an excessively small kernel size fails to capture the long-range temporal dependencies of EEG signals, while an overlarge kernel size tends to introduce noise or cause over-smoothing of features. K=45 achieves a favorable balance between modeling temporal dynamics and suppressing noise.

The influence of the number of filters *F* of the pointwise convolution in the spatial-temporal block on model accuracy is further investigated. On the BCIC IV 2a dataset, the accuracy continuously improves as *F* increases from 20 to 50, peaks at F=50, and then remains stable. On the BCIC IV 2b dataset, the accuracy rises rapidly to the highest point as *F* increases from 20 to 30 but gradually decreases as *F* continues to increase. This difference stems from the distinct number of channels between the two datasets. BCIC IV 2a has more channels, so a larger *F* can still ensure sufficient feature interaction without introducing significant redundancy. In contrast, BCIC IV 2b has relatively fewer channels. When *F* exceeds 30, the excessive expansion of the channel dimension intensifies feature redundancy, which reduces the discriminative ability of the model and eventually leads to accuracy degradation.

We also analyze the effect of the $L_{R-MMD}$ loss weight λ on model performance. Both datasets present a consistent rule that either an excessively large or small λ will cause performance degradation, but the optimal value varies with dataset characteristics. On the BCIC IV 2a dataset, the model achieves the highest classification accuracy when λ=1.0, while the optimal weight is λ=0.1 on the BCIC IV 2b dataset. When λ is too small, the constraint for domain alignment is insufficient to effectively eliminate the distribution shift between the source and target domains. When λ is too large, the excessively strong alignment constraint suppresses the model’s learning of the discriminative features unique to the target domain, reduces feature effectiveness, and impairs the generalization ability of the model.

## 6. Conclusions

The BARN-DA model proposed in this study effectively improves the decoding performance of MI-EEG signals. Specifically, the BACA module enhances channel-wise features by integrating frequency-band prior knowledge, while the MSKP module boosts the robustness of feature extraction. The design that combines Riemannian geometry with domain adaptation technology further narrows the domain distribution discrepancy, thereby endowing the model with excellent generalization ability. Tests conducted on four publicly available datasets validate that the BARN-DA framework attains average cross-session classification accuracies of 84.65% ± 8.97% for BCIC IV 2a, 89.19% ± 7.69% for BCIC IV 2b, and 61.76% ± 12.68% for SHU, alongside average cross-subject classification accuracies of 65.49% ± 11.64% for BCIC IV 2a, 78.78% ± 8.44% for BCIC IV 2b, and 78.14% ± 14.41% for BCIC III 4a. This work provides an efficient and reliable technical solution for the practical deployment of robust BCI systems.

## Figures and Tables

**Table 1:** Comparison of the proposed method with existing methods.

Method	Attention Mechanism	Frequency Band Aware	Riemannian Geometry	Domain Adaptation
CSP [16]	×	×	×	×
FBCSP [17]	×	✓	×	×
ConvNet [23]	×	×	×	×
EEGNet [24]	×	×	×	×
FBCNet [41]	×	✓	×	×
LMDA-Net [42]	✓	×	×	×
Wimpff et al. [29]	✓	×	×	×
ETCNet [28]	✓	×	×	×
SST-DPN [43]	✓	×	×	×
Fang et el. [44]	×	✓	✓	×
Jin et el. [45]	×	×	✓	×
Tensor-CSPNet [32]	×	×	✓	×
Graph-CSPNet [33]	×	×	✓	×
SecNet [34]	×	×	✓	×
Shi et el. [35]	×	×	✓	×
DJDAN [46]	×	×	×	✓
DAWD [38]	×	×	×	✓
DRDA [47]	×	×	×	✓
GAT [48]	✓	×	×	✓
EEG-DG [49]	×	×	×	✓
BARN-DA (Ours)	✓	✓	✓	✓

**Table 2:** Basic information of three datasets used in experiment.

Name	Channels	Subjects	Trials per Subject	Classes	Sampling Points	Experiment type
BCIC IV 2a	22	9	576	4	1000	Cross Session/Subject
BCIC IV 2b	3	9	720	2	1000	Cross Session/Subject
BCIC III 4a	118	5	280	2	350	Cross Subject
SHU	32	25	450~500	2	1000	Cross Session

**Table 3:** Training hyperparameter settings.

Hyperparameter	Value	Hyperparameter	Value
Optimizer	Adam	Batch size	64
Learning rate	0.002	Epochs (cross-session)	300
$\beta_{1}$ (Adam)	0.9	Epochs (cross-subject)	50
$\beta_{2}$ (Adam)	0.999	Weight decay	0

**Table 4:** Model parameter configuration for four datasets.

Description	Parameter	Specific Values on Different Datasets			
		BCIC IV 2a	BCIC IV 2b	BCIC III 4a	SHU
Temporal convolution filters number (ST-Block)	*D*	9	11	9	9
Temporal convolution kernel size (ST-Block)	*K*	45	45	45	45
Point-wise convolution filter number (ST-Block)	*F*	60	30	60	60
SPD matrix order	*F*	60	30	60	60
$L_{R-MMD}$	λ	1.0	0.1	1.0	1.0

**Table 5:** The cross-session performance comparison among different methods on dataset BCIC IV 2a.

Method	Subject Accuracy (%)									Avg	Std	*p*-Value
	S01	S02	S03	S04	S05	S06	S07	S08	S09			
CONVNET [23]	68.75	45.83	77.08	56.94	55.21	46.18	65.28	73.26	64.93	61.50	11.18	0.0020
EEGNET [24]	77.43	55.56	90.62	63.89	69.79	58.33	68.06	71.88	73.61	69.91	10.50	0.0020
DRDA ^†^ [47]	83.19	55.14	87.43	75.28	62.29	57.15	86.18	83.61	82.00	74.75	12.96	0.0020
DJDAN ^†^ [46]	86.46	68.75	93.06	85.42	72.57	63.54	**95.49**	85.76	83.68	81.52	10.94	0.0195
ATCNET [27]	79.51	58.68	89.24	69.79	69.44	62.15	86.11	84.03	75.69	74.96	10.71	0.0020
DAWD ^†^ [38]	83.29	63.97	90.30	76.94	69.34	60.08	89.31	82.35	82.81	77.60	10.85	0.0020
GAT ^†^ [48]	88.89	61.11	93.40	71.86	50.35	60.07	89.58	87.50	86.46	76.58	15.98	0.0178
CTNET [30]	80.90	58.68	84.38	76.39	70.49	65.28	82.64	78.82	72.92	74.50	8.52	0.0020
EEG-DG ^†^ [49]	**89.24**	64.93	94.79	**85.76**	68.75	61.46	95.14	**88.89**	**87.15**	81.79	13.06	0.1623
SST-DPN [43]	85.42	55.90	92.01	77.08	80.90	67.71	86.81	85.42	84.03	79.48	11.20	0.0020
BARN-DA	88.19	**69.10**	**96.18**	84.72	**81.94**	**73.26**	95.14	87.50	85.76	**84.65**	8.97	-

The symbol ^†^ denotes the experimental results used in original paper.

**Table 6:** The cross-session performance comparison among different methods on dataset BCIC IV 2b.

Method	Subject Accuracy (%)									Avg	Std	*p*-Value
	S01	S02	S03	S04	S05	S06	S07	S08	S09			
CONVNET [23]	72.81	61.43	75.94	95.94	90.62	83.75	87.50	89.69	81.56	82.14	10.64	0.0020
EEGNET [24]	71.56	69.64	81.25	97.50	81.56	83.44	89.38	94.37	84.38	83.68	9.31	0.0086
DRDA ^†^ [47]	81.37	62.86	63.63	95.94	93.56	88.19	85.00	**95.25**	90.00	83.98	12.67	0.0273
DJDAN ^†^ [46]	83.44	58.57	59.06	98.13	96.56	84.38	86.25	92.81	87.81	83.00	14.64	0.0371
ATCNET [27]	69.06	67.86	78.44	97.19	97.19	82.50	**92.81**	92.81	86.88	84.97	11.29	0.0273
DAWD ^†^ [38]	**84.66**	66.57	68.04	96.78	94.32	82.61	88.47	93.96	90.10	85.06	11.05	0.0645
GAT ^†^ [48]	84.58	61.67	60.83	**99.58**	87.50	**93.33**	85.42	95.00	**92.08**	84.44	13.98	0.2129
CTNET [30]	74.38	68.57	83.13	97.19	84.38	85.00	90.94	92.50	84.06	84.46	8.84	0.0059
EEG-DG ^†^ [49]	82.50	67.50	72.19	98.44	96.56	90.94	89.38	95.00	91.56	87.12	10.89	0.2480
SST-DPN [43]	79.37	64.64	80.00	96.88	96.56	85.00	88.44	90.00	85.00	85.10	9.89	0.0020
BARN-DA	82.19	**73.93**	**85.31**	97.50	**98.12**	92.19	91.87	92.50	89.06	**89.19**	7.69	-

The symbol ^†^ denotes the experimental results used in original paper.

**Table 7:** The cross-session performance comparison among different methods on dataset SHU.

Subject	Method Accuracy (%)					
	CONVNET [23]	EEGNET [24]	ATCNET [27]	CTNET [30]	SST-DPN [43]	BARN-DA
Sub 01	52.33	44.04	45.60	49.74	53.89	**59.07**
Sub 02	48.72	59.49	54.87	53.85	56.41	**67.69**
Sub 03	51.50	58.50	59.50	58.50	**60.00**	57.00
Sub 04	**58.00**	50.00	51.50	50.00	52.00	53.00
Sub 05	53.19	39.89	50.00	46.28	**56.38**	50.00
Sub 06	63.64	74.87	66.31	73.80	70.59	**81.28**
Sub 07	61.46	54.69	58.85	52.60	51.56	**69.27**
Sub 08	50.51	48.98	50.51	51.02	50.51	**52.55**
Sub 09	46.15	51.79	51.79	53.33	49.74	**55.90**
Sub 10	56.35	51.78	57.36	**61.93**	51.78	55.84
Sub 11	53.50	**56.50**	55.50	53.00	51.50	48.00
Sub 12	51.18	**54.12**	49.41	51.76	50.00	49.41
Sub 13	54.87	55.38	55.90	60.00	55.38	**73.33**
Sub 14	58.33	51.56	52.60	51.04	54.17	**59.38**
Sub 15	77.20	72.54	73.58	73.58	75.13	**79.27**
Sub 16	62.37	58.60	55.38	60.22	61.29	**67.20**
Sub 17	51.28	49.23	52.31	51.28	**53.33**	47.69
Sub 18	56.00	51.50	54.50	52.50	50.50	**58.50**
Sub 19	60.00	**66.49**	61.62	64.32	62.16	61.62
Sub 20	84.50	94.00	84.00	93.50	83.00	**96.50**
Sub 21	68.88	69.90	72.45	64.80	66.84	**83.16**
Sub 22	53.89	52.85	61.14	**64.77**	62.69	61.14
Sub 23	64.97	69.04	**73.60**	63.96	50.76	53.30
Sub 24	50.00	50.00	49.49	**57.07**	44.95	47.98
Sub 25	**56.38**	47.87	44.15	47.34	49.47	55.85
Avg	57.81	57.34	57.68	58.41	56.96	**61.76**
Std	8.89	11.64	9.70	10.47	9.00	12.68
*p*-value	0.0157	0.0044	0.0114	0.0272	0.0018	-

**Table 8:** The cross-subject performance comparison among different methods on dataset BCIC IV 2a.

Method	Subject Accuracy (%)									Avg	Std	*p*-Value
	S01	S02	S03	S04	S05	S06	S07	S08	S09			
CONVNET [23]	68.92	43.23	74.31	50.69	37.33	48.09	63.72	66.67	64.58	57.50	12.90	0.0020
EEGNET [24]	64.93	**51.22**	71.18	49.13	55.38	54.69	71.35	73.44	58.33	61.07	9.33	0.0098
ATCNET [27]	60.94	47.40	76.39	55.90	57.99	38.19	71.35	71.35	**67.19**	60.74	12.40	0.0195
CTNET [30]	68.58	49.83	73.44	**57.47**	44.10	51.74	70.83	75.35	66.67	62.00	11.44	0.0820
SST-DPN [43]	66.15	44.97	76.39	50.00	46.53	50.00	65.80	59.38	61.63	57.87	10.66	0.0020
BARN-DA	**69.44**	47.57	**82.29**	53.65	**60.59**	**57.47**	**74.48**	**77.95**	65.97	**65.49**	11.64	-

**Table 9:** The cross-subject performance comparison among different methods on dataset BCIC IV 2b.

Method	Subject Accuracy (%)									Avg	Std	*p*-Value
	S01	S02	S03	S04	S05	S06	S07	S08	S09			
CONVNET [23]	70.83	64.41	58.75	80.95	85.27	75.56	74.58	77.24	76.67	73.81	8.14	0.0020
EEGNET [24]	68.61	**70.29**	63.33	73.78	80.68	76.25	**86.94**	75.13	73.33	74.26	6.85	0.0195
ATCNET [27]	75.28	67.65	54.44	79.32	80.14	71.81	76.11	74.87	75.14	72.75	7.81	0.0020
CTNET [30]	74.44	69.41	61.39	85.00	84.86	77.78	82.64	77.24	75.69	76.50	7.63	0.0039
SST-DPN [43]	70.42	68.24	60.69	78.92	84.32	77.36	79.31	68.42	**77.92**	73.95	7.41	0.0059
BARN-DA	**77.36**	68.53	**63.61**	**88.65**	**87.30**	**83.33**	85.00	**78.29**	76.94	**78.78**	8.44	-

**Table 10:** The cross-subject performance comparison among different methods on dataset BCIC III 4a.

Method	Subject Accuracy (%)					Avg	Std	*p*-Value
	aa	al	av	aw	ay			
CONVNET [23]	77.50	81.07	51.43	83.93	54.64	69.71	15.44	0.0312
EEGNET [24]	70.71	70.00	59.29	61.43	56.43	63.57	6.45	0.0312
ATCNET [27]	70.36	70.00	57.86	61.43	53.93	62.71	7.31	0.0312
CTNET [30]	73.57	61.79	58.57	56.43	57.50	61.57	7.00	0.0312
SST-DPN [43]	65.36	74.29	54.29	54.64	57.86	61.29	8.52	0.0312
BARN-DA	**83.93**	**95.71**	**62.50**	**84.64**	**63.93**	**78.14**	14.41	-

**Table 11:** Ablation study of the proposed method on different datasets.

Dataset	Method	Avg. Accuracy (%)	Std	*p*-Value
BCIC IV 2a (cross-session)	w/o BACA	82.37	10.32	0.0195
	w/o MSKP	78.74	10.66	0.0039
	w/o $L_{R-MMD}$	79.40	10.75	0.0020
	w/o BACA & MSKP	76.62	12.01	0.0020
	BARN-DA	84.65	8.97	-
BCIC IV 2b (cross-session)	w/o BACA	88.15	8.97	0.0352
	w/o MSKP	87.66	8.29	0.0137
	w/o $L_{R-MMD}$	87.81	9.32	0.0391
	w/o BACA & MSKP	83.41	12.56	0.0020
	BARN-DA	89.19	7.69	-
BCIC IV 2a (cross-subject)	w/o BACA	63.31	10.99	0.0020
	w/o MSKP	58.24	11.83	0.0039
	w/o $L_{R-MMD}$	61.69	11.34	0.0020
	w/o BACA & MSKP	52.51	11.19	0.0039
	BARN-DA	65.49	11.64	-
BCIC IV 2b (cross-subject)	w/o BACA	78.61	7.97	0.2734
	w/o MSKP	75.09	9.29	0.0039
	w/o $L_{R-MMD}$	77.69	8.37	0.0273
	w/o BACA & MSKP	73.44	9.55	0.0020
	BARN-DA	78.78	8.44	-
BCIC III 4a (cross-subject)	w/o BACA	76.50	12.27	0.3125
	w/o MSKP	73.50	13.71	0.0312
	w/o $L_{R-MMD}$	70.07	12.79	0.0312
	w/o BACA & MSKP	74.71	14.51	0.0312
	BARN-DA	78.14	14.41	-
SHU (cross-session)	w/o BACA	59.82	11.27	0.1239
	w/o MSKP	59.74	11.91	0.0564
	w/o $L_{R-MMD}$	56.67	9.64	0.0033
	w/o BACA & MSKP	61.76	12.20	0.4772
	BARN-DA	61.76	12.68	-

**Table 12:** Comparison of parameters, computational complexity, and time cost of different models.

Dataset	Method	Params (B)	Flops (M)	Train Time (s)	Test Time (s)
BCIC IV 2a (cross-session)	CONVNET [23]	46,084	55.60	1523.88	0.046
	EEGNET [24]	3444	12.20	24.82	0.014
	ATCNET [27]	113,732	26.36	270.43	0.613
	CTNET [30]	152,684	35.94	57.19	0.023
	SST-DPN [43]	19,502	9.70	34.04	0.039
	BARN-DA	35,622	24.55	168.68	0.097
BCIC IV 2b (cross-session)	CONVNET [23]	10,802	7.78	8.83	0.006
	EEGNET [24]	2146	1.77	7.33	0.004
	ATCNET [27]	112,794	5.67	343.12	0.675
	CTNET [30]	150,722	9.34	36.05	0.009
	SST-DPN [43]	6493	1.49	13.75	0.012
	BARN-DA	9732	4.62	132.31	0.068
BCIC III 4a (cross-subject)	CONVNET [23]	191,282	100.07	76.12	1.148
	EEGNET [24]	3314	22.52	47.82	0.024
	ATCNET [27]	116,474	45.86	248.39	0.658
	CTNET [30]	154,522	59.57	107.73	0.037
	SST-DPN [43]	56,334	17.91	36.73	0.056
	BARN-DA	81,200	26.74	87.65	0.070
SHU (cross-session)	CONVNET [23]	57,202	81.37	1904.11	0.049
	EEGNET [24]	2610	17.55	40.17	0.016
	ATCNET [27]	113,722	37.25	307.85	0.434
	CTNET [30]	151,882	49.94	81.56	0.024
	SST-DPN [43]	19,804	14.02	50.53	0.039
	BARN-DA	37,360	29.94	193.16	0.071

## Data Availability

The BCI Competition IV 2a, 2b, BCI Competition III 4a, and SHU datasets utilized in this study are publicly accessible. They can be downloaded from the following websites: https://bnci-horizon-2020.eu/database/data-sets (accessed on 23 March 2026); https://www.bbci.de/competition/iii/ (accessed on 23 March 2026); https://figshare.com/articles/software/shu_dataset/19228725/1 (accessed on 23 March 2026). And the code is available at https://github.com/zhwangx/BARN-DA (accessed on 23 March 2026).
